# Trends in Sedentary Behavior, Physical Activity, and Motivation during a Classroom-Based Active Video Game Program

**DOI:** 10.3390/ijerph16162821

**Published:** 2019-08-07

**Authors:** You Fu, Ryan D. Burns, Emma Gomes, Amy Savignac, Nora Constantino

**Affiliations:** 1 School of Community Health Sciences, University of Nevada Reno, Reno, NV 89557, USA; 2 Department of Health, Kinesiology, and Recreation, University of Utah, Salt Lake City, UT 84112, USA

**Keywords:** school children, physical activity, psychosocial variables, exergaming

## Abstract

The purpose of this preliminary study was to investigate trends in children’s sedentary behavior (SB), physical activity (PA), and motivation during a 12 week classroom-based Active Video Game (AVG) program. A sample of 16 children, recruited from an elementary school, participated in AVG for 30 minutes per school day for 12 consecutive weeks. School day time in SB and PA, in addition to step counts, were assessed across 12 weeks using accelerometers and motivation was assessed via questionnaires. Mixed effects models with a quadratic time parameter were employed to examine time trends. A significant negative trend was observed for SB, while light and vigorous PA and step counts yielded positive trends until approximately 8–9 weeks where a quadratic inflection point was observed (*p* < 0.001). Regarding motivational variables, enjoyment and social support from teachers significantly increased across 12 weeks (*p* < 0.05). A 12 week classroom AVG program yielded a positive trend in school day light and vigorous PA and step counts, and a negative trend in SB until 8–9 weeks into the program. This study supports the use of low-cost classroom-based AVG programs to improve children’s physical and mental health, but favorable PA trends were attenuated past 8–9 weeks.

## 1. Introduction

In the US, the prevalence of children affected by overweight and obesity has more than tripled since the 1970s [1]. Recent data show that nearly one in every five school-age children and young people (aged 6 to 19 years) suffer from obesity in the U.S. [2]. Among the many factors that contribute to childhood obesity is physical inactivity [3,4,5]. There is evidence that about 80% of school children do not achieve daily physical activity (PA) recommendations and a majority of children exceed sedentary behavior (SB) guidelines by spending more than 7.5 hours per day in front of a screen (e.g., TV and computer) [6,7,8]. It is estimated that 95% of American children attend school; therefore, the academic classroom is an important venue to help children accumulate and meet daily PA guidelines.

Unlike the conventional sedentary video games that may lead to sedentary lifestyles [9], active video games (AVGs) represent a variety of video games that require bodily movement and PA while playing, and have been increasingly used to promote PA and health in school settings [10]. A growing body of evidence suggests that AVGs have the potential to improve health behaviors (e.g., physical activity, physical fitness, motivation, motor skills) in youth [11,12,13,14]. Mechanisms to achieve these benefits include improvements in motivation (e.g., enjoyment, self-efficacy, perceived competence, and social support) that capture and sustain interest in PA [10]. The empirical literature further indicates that AVGs could reduce children’s SB and require energy expenditure comparable to light-to-moderate intensity exercise [10]. Furthermore, certain types of AVGs can yield as much energy expenditure as jogging or brisk walking [15].

The positive impacts of AVGs on SB, PA, and motivation have been well documented, and researchers have been trying to seek ways in which AVGs can be a desirable alternative to physical education and/or a daily PA option during recess [16,17,18,19]. Children’s outdoor play is associated with increased levels of activity and positive mood during school hours [20]. Due to its indoor and active nature, classroom-based AVGs may be used as an appropriate expenditure-increasing solution and well-being promotion approach when outdoor activities are not accessible for school children during inclement weather and winter seasons. However, investigations in the field have primarily been conducted in laboratory settings using small samples with cross-sectional data or using short-duration interventions [11,13,17]. Knowledge on the longitudinal changes and tendency in children’s health behaviors when participating in AVGs during a typical school semester is scarce. Moreover, most existing research has employed interactive AVGs that only allow 2–4 participants to play at a time, which also require certain gaming consoles and video cameras to facilitate interactions [12,16,18,21]. It is imperative to investigate the effectiveness of AVGs on children’s objective and longitudinal SB, PA, and motivation in an academic classroom setting, provided that AVG stations are easy to set up with no bulky cords in the space and that all children in the classroom have the opportunity to play the game at the same time. Therefore, the purpose of this preliminary study was to investigate the linear and non-linear trends of children’s objective SB, PA, and motivation during a classroom-based AVG program over 12 consecutive weeks.

## 2. Materials and Methods

### 2.1. Participants

Participants were a convenience sample of 16 elementary school students (mean age = 7.1 ± 0.7 years; six girls, 10 boys) in one classroom recruited from a public “Zoom” school located in the western U.S. Zoom schools enroll a large proportion of children who have limited English speaking proficiency and poor academic performance. Extra financial funding, tutoring programs, and academic services are supplemented at every Zoom school to improve English proficiency and academic achievements. The public Zoom school where the current study was conducted did not offer physical education to its students. Among all the participants, six students were of Hispanic ethnicity, six students were Caucasian, two were African American, and two were Asian American.

In this study, elementary school students were recruited because they were able to comprehend the rules and perform the AVGs. The inclusion criteria were children who were (1) enrolled in the participating elementary school, (2) aged 6–7 years, (3) without any diagnosed physical or mental disabilities as reported by school records, and (4) able to provide parental consent and child assent. Prior to data collection, written consent from the participants’ parent or guardian was obtained, and the University Institutional Review Board approved the study (IRB number: 1334451-3).

### 2.2. Procedures

This study employed a longitudinal repeated measure design. An AVG program was incorporated into the students’ daily classroom routine with support from school administrators and the classroom teacher during the spring semester of 2019. Specifically, children played a series of AVGs (e.g., Adventure to Fitness, GoNoodle, and Cosmic Kids Yoga) in their classroom over three separate 10 minute sessions daily (Monday–Friday) for 12 consecutive weeks, supervised by their full-time classroom teacher. In each 10 minute AVG session, children played 5 minutes of “GoNoodle”, then 2–3 minutes of “Adventure to Fitness,” and ended with 2–3 minutes allocated to “Cosmic Kids Yoga,” which was used as a cool-down activity. The games in each section were selected by the classroom teacher. “GoNoodle” is an online AVG program that provides children with PA and exercise options, such as dance, aerobic workouts, race competitions, and locomotor skill games. “Adventure to Fitness” includes active video clips while taking children on an adventure with various scenarios. The AVGs in each program tend to have a similar nature regarding the intensity, difficulty, and category. One AVG station included a computer, a projector, and a screen, and was set up in the classroom for all the children to play at the same time.

### 2.3. Measures

Physical activity: Children’s school day (9 am–3 pm) SB, light PA, moderate PA, vigorous PA, and step counts were assessed weekly over 12 consecutive weeks using ActiGraph wGT3X-BT accelerometers (ActiGraph, FL, USA), which were attached on the right side of each child’s waist. Activity counts were collected at 15 s epochs at 100 Hz, processed using counts per minute (CPM) cut-points (SB = 0–100 CPM; light PA = 101–2295 CPM; moderate to vigorous PA = 2296–4011 CPM) [22]. Outcome variables were the weekly means of the step counts and the time percentages of SB, light PA, moderate PA, and vigorous PA.

Motivation: Across the intervention period, motivation was assessed three times (week 1, week 6, and week 12) using identical self-report questionnaires, totaling 22 items across four domains, including perceived competence (six items; Perceived Competence Scale for Children) [23], enjoyment (four items; Sport Enjoyment Scale) [24], self-efficacy (six items) [25], and social support from teachers and peers (six items; Physical Activity Social Support Scale) [26]. The average score across items within each domain was used for data analysis. All sub-scales were determined to have acceptable internal consistency.

### 2.4. Statistical Analysis

Differences between sexes at baseline were examined using independent t-tests. To examine weekly (time) trends in SB, time in specific PA intensities, step counts, and specific motivational variables, general linear mixed effects models were employed using random intercepts and slopes. Unstructured covariance was used across the random effects with robust standard errors employed at the student level. The primary predictor variable was time (in weeks). To determine whether the 12 week time trends were non-linear for SB and PA, a quadratic time predictor was entered into each model. Because the motivational variables were collected across only three time-points, only the linear time predictor was examined for these outcomes. Effect sizes were calculated using Cohen’s d where d < 0.20 indicates a small effect size, d = 0.50 indicates a medium effect size, and d > 0.80 indicates a large effect size [27]. All motivational pair-wise comparisons were in relation to baseline scores. Alpha level was set at *p* < 0.05 and all analyses were carried out using the STATA v15.0 statistical software package (College Station, TX, USA).

## 3. Results

Descriptive statistics are reported in Table 1. Girls reported higher social support from teachers than boys at baseline (*p* = 0.040, d = 1.0). Results from the general linear mixed effects models are communicated in Table 2.

**Figure 1:**
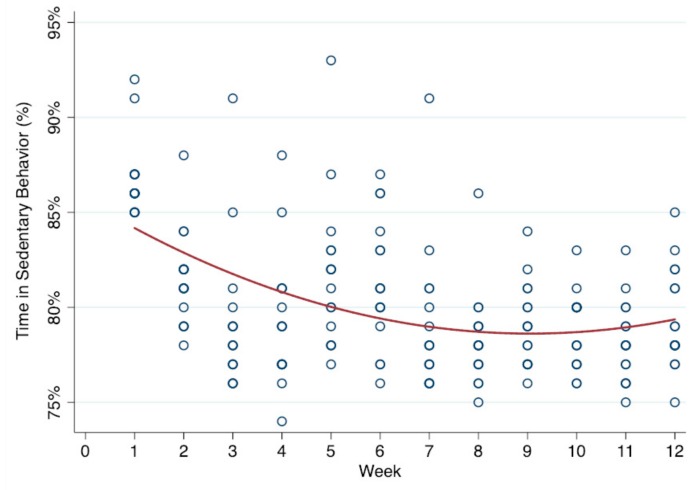
Quadratic time trend for sedentary behavior (%) across weeks.

**Figure 2:**
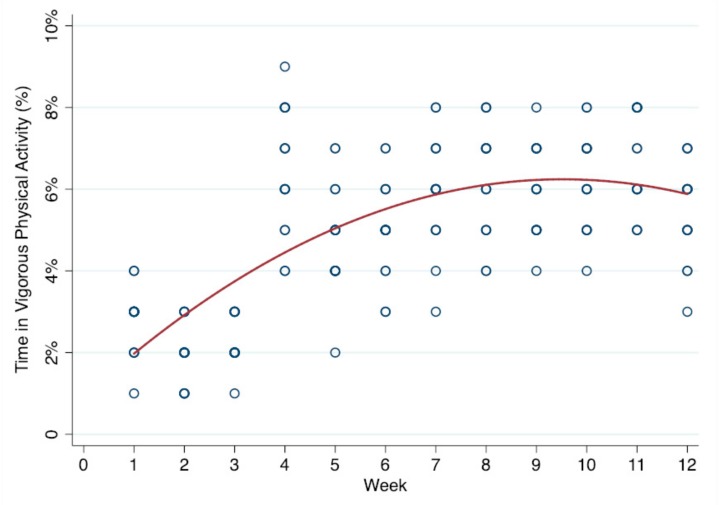
Quadratic time trend in vigorous physical activity (%) across weeks.

**Figure 3:**
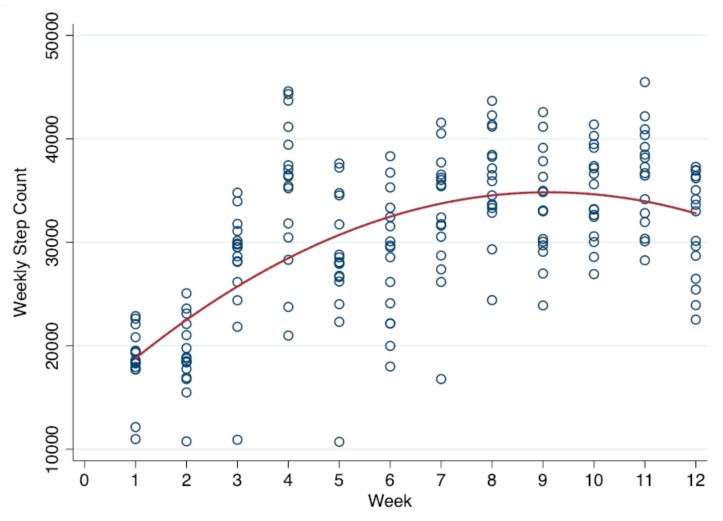
Quadratic time trend in step counts across weeks.

There were significant time trends for SB, light PA, vigorous PA, step counts, enjoyment, and social support from teachers (*p* < 0.001). Specifically, SB tended to decrease and time spent in light PA, vigorous PA, and step counts tended to increase. Moderate PA did not significantly change across the 12 weeks. Additionally, the significant trends also yielded a statistically significant quadratic time variable (*p* < 0.001), indicating that trends across weeks were non-linear with a point of inflection observed at approximately 8–9 weeks. Figure 1, Figure 2 and Figure 3 display the significant quadratic time trends for SB, vigorous PA, and step counts, respectively.

Concerning the motivational variables, both enjoyment and social support from teachers increased across the program. Enjoyment increased from 3.6 (SD = 0.9) at week 1 to 3.9 (SD = 0.7) at week 6 (*p* = 0.232, d = 0.31) and to 4.1 (SD = 0.8) at week 12 (*p* = 0.047, d = 0.56). Social support from teachers increased from 2.5 (SD = 0.7) at week 1 to 2.9 (SD = 0.9) at week 6 (*p* = 0.275, d = 0.27) and to 3.5 (SD = 0.4) at week 12 (*p* < 0.001, d = 1.4).

## 4. Discussion

The results suggest that the AVG program yielded a negative trend in SB, while it yielded a positive trend in PA in various intensities and motivation over 12 consecutive weeks. In particular, it was vigorous PA rather than moderate PA that increased across time, which was also reflected in the fact that children increased their steps per school week by about 4446. Interestingly, another important finding of the current study was that these favorable trends were observed until weeks 8–9, as seen in Figure 1, Figure 2 and Figure 3. In other words, there was an inflection point after which the positive effects of the intervention were attenuated. The flattening of the trend curve at weeks 8–9 could possibly be due to the characteristics of the AVG program itself. For instance, certain games (e.g., Cosmic Kids Yoga) employed in this study were presented with low intensity and short durations. AVG programs may also have a ceiling effect on young children’s PA, meaning that the given time, frequency, and duration of the intervention could only yield limited PA improvements. Therefore, in order to improve PA above and beyond what has already been observed, the characteristics (e.g., frequency, duration, games options, and intense exercises) of the AVG program would need to be changed to further affect PA volume and energy expenditure. 

In addition to the aforementioned reasoning for the observed quadratic time trends, the attenuated improvements in SB and PA past 8–9 weeks may also be a result of the type of PA assessment used in this study. Accelerometry only captures ambulatory PA; therefore, accelerometers may not have detected upper body movements and/or muscle strengthening activities that involve resistance. For instance, children only moved their upper bodies and limbs during some of the dancing games in GoNoodle and certain Yoga movements; in this case, the accelerometers would not capture these types of behaviors. Future research should be conducted using assessments that could detect and evaluate the subject’s upper body movements. 

Nevertheless, the lack of improvement in vigorous PA and step counts past weeks 8–9 was probably not due to the level of enjoyment or social support from teachers, as these motivational constructs increased significantly and consistently across time. These findings are soundly echoed by previous studies. For example, Gao et al. [20] examined the effect of AVGs on elementary school children’s objective SB, PA, and energy expenditure over 18 weeks, and positive impacts on these outcomes were reported. Furthermore, the results are similar to those of Staiano et al. [28] in that it was suggested that a 12 week AVG program significantly increased school students’ self-efficacy and enjoyment, and positive effects of AVGs were found on those young individuals’ PA and SB.

The strengths of this preliminary study include: (1) the employment of a classroom-based AVG program that was supervised by the children’s classroom teacher, (2) the objective assessment of SB, PA, and step counts, (3) the analysis of specific intensities of PA prospectively observed across 12 weeks, and (4) the collection of salient motivational variables that may have influenced the trends in SB and PA across 12 weeks. However, the current study is not without limitations. First, as the study was conducted within one classroom with 16 participants, it is therefore difficult to generalize the findings. The small sample is likely statistically underpowered; however, given the difficulty in collecting longitudinal accelerometer data on young children over a period of 12 consecutive weeks, the obtained data have merit.

## 5. Conclusions

In conclusion, the results of this preliminary study contribute to the existing literature on the benefits of AVGs in promoting young children’s PA and motivation. The employment of the classroom-based AVGs tended to reduce SB and increase PA up to about 8–9 weeks. Past 8–9 weeks, further modification of the program may be needed to continually improve PA.

## Figures and Tables

**Table 1:** Descriptive statistics for the total sample and within sex groups at baseline (week 1; means and standard deviations).

Outcome	Sample (n = 16)	Girls (n = 6)	Boys (n = 10)
SB Time (%)	86.6% (2.0%)	86.7% (2.2%)	86.7% (2.0%)
Light PA (%)	7.7% (1.1%)	7.5% (1.0%)	7.8% (1.1%)
Moderate PA (%)	2.7% (0.7%)	2.8% (0.8%)	2.6% (0.7%)
Vigorous PA (%)	2.8% (0.8%)	2.5% (0.8%)	3.0% (0.7%)
Weekly Steps	18,555 (3218)	17,829 (3691)	18,992 (3022)
Perceived Competence (1–4 Scale)	3.1 (0.6)	2.9 (0.6)	3.2 (0.6)
Enjoyment (1–4 Scale)	3.6 (0.9)	3.2 (1.1)	3.9 (0.8)
Self-Efficacy (1–5 Scale)	3.6 (1.0)	3.1 (1.4)	3.9 (0.6)
Social Support from Teachers (1–4 Scale)	2.5 (0.7)	2.9 ^†^ (0.6)	2.3 (0.5)
Social Support from Peers (1–4 Scale)	2.9 (0.8)	2.9 (0.9)	2.9 (0.9)

Note: SB stands for sedentary behavior; PA stands for physical activity; bold and ^†^ denote statistical differences between sexes, *p* < 0.05.

**Table 2:** Parameter estimates from the general linear mixed-effects models.

Outcome	Time Predictor (in Weeks)	b-coefficient	95% CI (%)	*P*-value
SB Time (%)	Time	−1.6% ^†^	−2.1–1.0%	<0.001
	Time ^ 2	0.09% ^†^	0.04–0.12%	<0.001
Light PA (%)	Time	0.7%	0.4–0.9%	<0.001
	Time ^ 2	−0.03% ^†^	−0.05–0.01%	<0.001
Moderate PA (%)	Time	−0.16%	−0.4–0.1%	0.234
	Time ^ 2	0.0%	−0.01–0.02%	0.551
Vigorous PA (%)	Time	1.1% ^†^	0.9–1.3%	<0.001
	Time ^ 2	−0.06% ^†^	−0.07–0.04%	<0.001
Weekly Steps	Time	4446 ^†^	3495–5398	<0.001
	Time ^ 2	−244 ^†^	−315–172	<0.001
Perceived Competence (1–4 Scale)	Time	0.16	−0.03–0.35	0.098
Enjoyment (1–4 Scale)	Time	0.24 ^†^	0.00–0.48	0.047
Self-Efficacy (1–5 Scale)	Time	0.27	−0.05–0.58	0.101
Social Support from Teachers (1–4 Scale)	Time	0.51 ^†^	0.29–0.73	<0.001
Social Support from Peers (1–4 Scale)	Time	−0.16	−0.38–0.07	0.174

Note: SB stands for sedentary behavior; PA stands for physical activity; bold and ^†^ denote statistical significance, *p* < 0.05.

## References

[1] Fryar C.D., Carroll M.D., Ogden C.L. (2014). Prevalence of overweight and obesity among children and adolescents aged 2–19 years: United States, 1963–1965 through 2011–2012. Health E Stats.

[2] Hales C.M., Carroll M.D., Fryar C.D., Ogden C.L. (2017). Prevalence of obesity among adults and youth: United States, 2015–2016. NCHS Data Brief.

[3] Ogden C.L., Carroll M.D., Curtin L.R., Lamb M.M., Flegal K.M. (2010). Prevalence of high body mass index in US children and adolescents, 2007–2008. JAMA.

[4] Skinner A.C., Skelton J.A. (2014). Prevalence and trends in obesity and severe obesity among children in the United States (1999–2012). JAMA Pediatr..

[5] Eisenburg L.K., Can Wijk K.J.E., Liefbroer A.C., Smidt N. (2017). Accumulation of adverse childhood events and overweight in children: A systematic review and meta-analysis. Obesity.

[6] National Physical Activity Plan Alliance (2016). 2016 US Report Card on Physical Activity for Children and Youth.

[7] Troiano R.P., Berrigan D., Dodd K.W., Masse L.C., Tilert T., Mc Dowell M. (2008). Physical activity in the United States measured by accelerometer. Med. Sci. Sports Exerc..

[8] Rideout V.J., Foehr U.G., Roberts D.F. (2010). Generation M2: Media in the Lives of 8 to 18-Year-Olds.

[9] Gordan-Larsen P., Adair L.S., Popkin B.M. (2002). Ethnic differences in physical activity and inactivity patterns and overweight status. Obes. Res..

[10] Gao Z. (2017). Fight fire with fire? Promoting physical activity and health through active video games. J. Sport Health Sci..

[11] Chen H., Sun H.C. (2017). Effects of active videogame and sports, play, and active recreation for kids physical education on children’s health-related fitness and enjoyment. Games Health J..

[12] Pasco D., Roure C., Kermarrec G., Pope Z., Gao Z. (2017). The effects of a bike active video game on players’ physical activity and motivation. J. Sport Health Sci..

[13] Gao Z., Chen S., Pasco D., Pope Z. (2015). A meta-analysis of active video games on health outcomes among children and adolescents. Obes. Rev..

[14] Fu Y., Burns R. (2018). Effect of an active video gaming classroom curriculum on health-related fitness, school day step counts, and motivation in sixth graders. J. Phys. Act. Health.

[15] Gao Z., Chen S. (2014). Are field-based exergames useful in preventing childhood obesity? A systematic review. Obe. Rev..

[16] Gao Z., Zhang T., Stodden D. (2013). Children’s physical activity levels and psychological correlates in interactive dance versus aerobic dance. J. Phys. Act. Health.

[17] Gao Z. (2012). Motivated but not active: The dilemmas of incorporating interactive dance into gym class. J. Phys. Act. Health.

[18] Norris E., Hamer M., Stamatakis E. (2016). Active video games in schools and effects on physical activity and health: a systematic review. J. Pediatr.

[19] Ennis C.D. (2013). Implications of exergaming for the physical education curriculum in the 21st century. J. Sport Health Sci..

[20] Gao Z., Pope Z., Lee J.E., Stodden D., Roncesalle N., Pasco D., Charles C., Feng D. (2017). Impact of exergaming on young children’s school day energy expenditure and moderate-to-vigorous physical activity levels. J. Sport Health Sci..

[21] Beets M.W., Randy V., Stanley C., Kenneth H.P., Bradley J.C. (2007). Parent’s Social Support for Children’s Outdoor Physical Activity: Do Weekdays and Weekends Matter?. Sex Roles.

[22] Evenson K.R., Catellier D.J., Gill K., Ondrak K.S., McMurray R.G. (2008). Calibration of two objective measures of physical activity for children. J. Sports Sci..

[23] Harter S. (1978). Effectance Motivation Reconsidered. Toward a Developmental Model. Hum. Dev..

[24] Scanlan T.K., Carpenter P.J., Schmidt G.W., Simons J.P., Keeler B. (1993). An introduction to the sport commitment model. J. Sport Exerc. Psycho..

[25] Gao Z., Newton M., Carson R.L. (2008). Students’motivation, physical activity Levels and health-related physical fitness in middle school physical education. MGRJ.

[26] Ommundsen Y., Page A., Ku P.W., Cooper A.R. (2008). Cross-cultural, age and gender validation of a computerised questionnaire measuring personal, social and environmental associations with children’s physical activity: The European Youth Heart Study. Int. J. Behav. Nutrit. Phys. Act.

[27] Cohen J., Hillsdale N.J.L. (1988). Statistical Power Analysis for the Behavioral Sciences.

[28] Staiano A.E., Beyl R.A., Hsia D.S., Katzmarzyk P.T., Newton R.L. (2017). Twelve weeks of dance exergaming in overweight and obese adolescent girls: Transfer effects on physical activity, screen time and self-efficacy. J. Sport Health Sci..

